# Body mass index, physical activity, and dietary behaviors among members of an urban community fitness center: a questionnaire survey

**DOI:** 10.1186/1471-2458-7-181

**Published:** 2007-07-26

**Authors:** Kimberly A Kaphingst, Gary G Bennett, Glorian Sorensen, Karen M Kaphingst, Amy E O'Neil, Kyle McInnis

**Affiliations:** 1 Center for Community-Based Research, Dana-Farber Cancer Institute, Boston, MA 02115, USA; 2 Department of Exercise and Health Services, University of Massachusetts-Boston, Boston, MA 02115, USA

## Abstract

**Background:**

Development of effective behavioral interventions to promote weight control and physical activity among diverse, underserved populations is a public health priority. Community focused wellness organizations, such as YMCAs, could provide a unique channel with which to reach such populations. This study assessed health behaviors and related characteristics of members of an urban YMCA facility.

**Methods:**

We surveyed 135 randomly selected members of an urban YMCA facility in Massachusetts to examine self-reported (1) physical activity, (2) dietary behaviors, (3) body mass index, and (4) correlates of behavior change among short-term (i.e., one year or less) and long-term (i.e., more than one year) members. Chi-square tests were used to assess bivariate associations between variables, and multivariate linear regression models were fit to examine correlates of health behaviors and weight status.

**Results:**

Eighty-nine percent of short-term and 94% of long-term members reported meeting current physical activity recommendations. Only 24% of short-term and 19% of long-term members met fruit and vegetable consumption recommendations, however, and more than half were overweight or obese. Length of membership was not significantly related to weight status, dietary behaviors, or physical activity. Most respondents were interested in changing health behaviors, in the preparation stage of change, and had high levels of self-efficacy to change behaviors. Short-term members had less education (p = 0.02), lower household incomes (p = 0.02), and were less likely to identify as white (p = 0.005) than long-term members. In multivariate models, females had lower BMI than males (p = 0.003) and reported less physical activity (p = 0.008). Physical activity was also inversely associated with age (p = 0.0004) and education (p = 0.02).

**Conclusion:**

Rates of overweight/obesity and fruit and vegetable consumption suggested that there is a need for a weight control intervention among members of an urban community YMCA. Membership in such a community wellness facility alone might not be sufficient to help members maintain a healthy weight. The data indicate that YMCA members are interested in making changes in their dietary and physical activity behaviors. Targeting newer YMCA members might be an effective way of reaching underserved populations. These data will help inform the development of a weight control intervention tailored to this setting.

## Background

Overweight and obesity have reached epidemic proportions in the United States, due mainly to consumption of high-calorie diets and low levels of physical activity [1]. The rate at which prevalence of obesity is growing is of particular concern among certain racial and ethnic minority populations, such as African Americans and Hispanics, in which the prevalence has increased rapidly over the last decade [2,3]. The development of behavioral interventions that target weight control and physical activity among members of underserved communities is critical to helping individuals meet existing behavioral recommendations and to reversing these trends [3].

Community focused wellness organizations such as YMCAs might provide a unique channel with which to reach underserved populations with community-based interventions that promote healthy lifestyles. YMCAs have a diverse membership with respect to age, race/ethnicity, income, and education. In addition, with about 2,500 YMCA facilities in the United States, the organization has a strong potential to reach a large number of individuals. Currently, there are approximately 20 million YMCA members, with about equal proportions of men and women [4]. YMCAs are accessible to traditionally underserved populations, since they are found in most urban areas throughout the U.S. and offer reduced-rate or free memberships based on ability to pay. Although many YMCAs offer health and wellness programs, the need among YMCA members for interventions focused on weight control and related health behaviors has not been systematically examined.

As part of exploring this need, we assessed self-reported physical activity, dietary behaviors, and rates of overweight and obesity among members of an urban YMCA in central Massachusetts which serves a sociodemographically diverse population. Other characteristics of members that might affect need for and responsiveness to weight control interventions (e.g., self-efficacy, motivation to change) were also examined. We were particularly interested in comparing the characteristics of short-term and long-term YMCA members to inform decisions about the appropriate target population for an intervention. We hypothesized that a smaller percentage of short-term members would be meeting current physical activity, dietary, and weight recommendations compared to long-term members, who might have more established fitness and wellness behavioral patterns. The purpose of this study was to characterize health behaviors and related characteristics among YMCA members as part of examining the need for a weight control intervention in this population.

## Methods

### Sample

We surveyed a randomly selected sample of members of the YMCA of Greater Worcester-Central Branch in Massachusetts. We first drew a random sample of 304 members ages 18 years and older from the list of currently enrolled members, a sample of approximately 10% of the current membership. Potential respondents were sent a letter addressed from the executive director of the YMCA facility explaining the study and giving them an opportunity to opt out of further contact. Members who did not respond to the letter within two weeks were contacted by telephone and asked to complete a 20-minute survey by telephone or in person at the YMCA facility. Mail and telephone contact information were drawn from current YMCA membership records. All respondents completed the informed consent process and the survey by telephone. We excluded individuals who did not speak English (n = 12), were no longer members of the YMCA (n = 1), had moved out of the area (n = 7), or for whom we could not obtain current contact information through the telephone company or electronic databases (n = 56); 228 individuals were eligible to participate. Of these, 41 (18%) refused to participate. We made at least six attempts to call each individual and sent a follow-up letter, but were unable to contact an additional 52 (23%) individuals. 135 members completed the survey, for a response rate among eligible individuals of 59.2%. The Office for the Protection of Research Subjects at the Dana-Farber Cancer Institute and the Institutional Review Board at the University of Massachusetts-Boston approved this study.

### Measures

#### Physical activity

We used the Short Form of the International Physical Activity Questionnaire (IPAQ) to assess physical activity behaviors. The IPAQ measures time spent in vigorous and moderate physical activities [5].

#### Dietary behaviors

We measured fruit and vegetable consumption using a screener developed for the National Cancer Institute (NCI)'s 5-A-Day for Better Health studies [6]. We recoded responses to equivalent servings per day and then summed to obtain total fruit and vegetable servings consumed per day. We also asked respondents how many days per week on average they took a multivitamin.

#### Cigarette smoking

Smoking status was assessed using standard NCI questions concerning lifetime smoking (e.g., having smoked at least 100 cigarettes in lifetime) and current smoking [7].

#### Motivation to change

We used separate sets of items to assess motivation to change physical activity and dietary behaviors further (e.g., becoming more physically active) using measures previously validated for physical activity [8,9] and adapted for dietary behaviors (e.g., "In the next six months, are you seriously thinking about becoming more physically active," with response options of "yes," "no," and "maybe"). These measures were previously developed and validated as part of two large cancer prevention intervention trials [10-12]. Respondents were classified into three groups: (1) precontemplation (i.e., not considering behavior change in the next six months); (2) contemplation (i.e., considering behavior change within the next six months, but not the next 30 days); and (3) preparation (i.e., planning behavior change within the next 30 days).

#### Self-efficacy

We used three-point Likert scale items to assess self-efficacy [13] to change physical activity and dietary behaviors (e.g., "If you thought you needed to improve your physical activity habits, how sure are you that you could do something about it in the next 30 days," with response options of "sure," "maybe," and "not at all sure") [10-12].

#### YMCA participation

We asked about length of YMCA membership and use of facilities for exercise within the last three months.

#### Interest in changing behaviors

We measured interest in changing physical activity and dietary behaviors using two four-point Likert scale items (e.g., "How interested are you in changing your physical activity habits," with response options from "not at all interested" to "very interested") [10]. We also assessed interest in losing weight, the amount of weight respondents wished to lose, and interest in participating in a weight control program at the YMCA.

#### Sociodemographics

Respondents reported their gender, height and weight (used to calculate body mass index (BMI) as kg/m^2^), date of birth, level of education completed, racial and ethnic background, employment status, household composition, and annual household income. For race/ethnicity, we coded respondents who reported being of Hispanic or Latino origin as Hispanic regardless of other racial or ethnic groups mentioned. Individuals with a self-reported BMI of between 25.0 kg/m^2 ^and 29.9 kg/m^2 ^were classified as overweight; those with a BMI of 30.0 kg/m^2 ^or greater were classified as obese.

### Analysis

We performed the data analysis using SAS 9.1 for Windows (SAS Institute, Cary, NC). We examined descriptive statistics for all variables. Bivariate associations between variables were assessed with chi-square tests. We fit three multivariate linear regression models to examine correlates of BMI, minutes of physical activity per week, and servings of fruits and vegetables per day using forward and backward stepwise regression procedures. Significant correlates (p < 0.05) were retained in the multivariate models. Missing data were excluded from the analyses.

## Results

We report the characteristics of the respondents divided into two categories, respondents who had been members for one year or less (i.e., short-term members) and respondents who had been members for more than one year (i.e., long-term members). Forty-six (34%) respondents were short-term members; 89 (66%) were long-term members. Fifty-six (63%) of long-term members had been members for five or more years.

As shown in Table 1, the majority of respondents (62%) were female; there were significantly more women among short-term (72%) than long-term (51%) members (p = 0.02). Thirty-one percent of respondents overall and 47% of short-term members had an annual household income of under $40,000; income was significantly associated with length of membership (p = 0.02). Long-term members had higher mean levels of education (p = 0.02). Fifty-four percent of short-term members and 80% of long-term members described themselves as white; the relationship between race/ethnicity and length of membership was significant (p = 0.005). Forty-one percent of respondents overall were ever smokers; 10% were current smokers. Current smokers were overrepresented among short-term members compared to long-term members, although this difference was not statistically significant.

**Table 1:** Differences in sociodemographic characteristics of a randomly selected sample of YMCA members by length of membership (n = 135)

Sociodemographic characteristic	Member 1 year or less (n = 46) (%)^a^	Member more than 1 year (n = 89) (%)^a^
*Gender*^*b*^		
Male	13 (28%)	44 (49%)
Female	33 (72%)	45 (51%)
		
*Education*^*b*^		
Some high school	4 (9%)	0 (0%)
Completed high school	7 (16%)	9 (10%)
Vocational/trade school	4 (9%)	4 (4%)
Some college	8 (18%)	13 (15%)
Associate degree	4 (9%)	6 (7%)
Bachelor degree	11 (24%)	24 (27%)
Post graduate degree	7 (16%)	33 (37%)
		
*Race/ethnicity*^c, d^		
Black	7 (14%)	6 (6%)
Hispanic	10 (20%)	5 (5%)
White	27 (54%)	77 (80%)
Other	6 (12%)	8 (8%)
		
*Currently employed*	30 (65%)	67 (75%)
		
*Household income*^*b*^		
Under $20,000	8 (21%)	11 (14%)
$20,000–$39,999	10 (26%)	8 (10%)
$40,000–$59,999	8 (21%)	14 (18%)
$60,000–$79,999	7 (18%)	13 (16%)
$80,000 or above	6 (15%)	33 (42%)
		
*Smoking history*		
Ever smoker	16 (35%)	40 (45%)
Current smoker	8 (17%)	6 (8%)
		
*Age*		
29 or younger	14 (30%)	4 (4%)
30–39	12 (26%)	18 (20%)
40–49	7 (15%)	33 (37%)
50–59	6 (13%)	14 (16%)
60 or older	7 (15%)	20 (22%)

a) Percentage of available cases. Percentage values might not total 100 due to rounding.

b) p = 0.02; null hypothesis of no difference in characteristic by length of membership.

c) Respondents could indicate more than one racial/ethnic category.

d) p = 0.005; null hypothesis of no difference in characteristic by length of membership.

Most short-term (89%) and long-term members (94%) reported obtaining at least 150 minutes per week of vigorous or moderate physical activity, as shown in Table 2. The majority of both short-term and long-term members reported being very or somewhat interested in changing physical activity (76% and 73%, respectively) and were in the preparation stage of change (80% and 70%, respectively). The majority of both groups had a high level of self-efficacy to change physical activity (67% of short-term and 83% of long-term members).

**Table 2:** Differences in health behaviors and weight status in a randomly selected sample of YMCA members by length of membership (n = 135)

***Health behavior *** Variable	*Member 1 year or less (n = 46) (%)*^*a*^	*Member more than 1 year (n = 89) (%)*^*a*^
**Physical activity**		
150 minutes/week or greater	41 (89%)	84 (94%)
		
*Interest in changing physical activity*		
Very interested	28 (61%)	44 (49%)
Somewhat interested	7 (15%)	21 (24%)
A little bit interested	5 (11%)	6 (7%)
Not at all interested	6 (13%)	18 (20%)
		
*Stages of change for physical activity*		
Preparation	37 (80%)	61 (70%)
Contemplation	2 (4%)	5 (6%)
Precontemplation	7 (15%)	21 (24%)
		
*Self-efficacy for changing physical activity*		
Sure	31 (67%)	73 (83%)
Maybe	12 (26%)	12 (14%)
Not at all sure	3 (7%)	3 (3%)
		
**Dietary**		
5+ servings of fruits and vegetables per day	11 (24%)	17 (19%)
		
*Multivitamin use*^*b*^		
7 days/week	13 (28%)	46 (52%)
1–6 days/week	9 (20%)	8 (9%)
Never	24 (52%)	35 (39%)
		
*Interest in changing dietary habits*		
Very interested	17 (37%)	31 (35%)
Somewhat interested	16 (35%)	26 (29%)
A little bit interested	3 (7%)	16 (18%)
Not at all interested	10 (22%)	16 (18%)
		
*Stages of change for dietary habits*		
Preparation	27 (59%)	58 (66%)
Contemplation	4 (9%)	3 (3%)
Precontemplation	15 (33%)	27 (31%)
		
*Self-efficacy for changing dietary habits*		
Sure	29 (64%)	65 (74%)
Maybe	10 (22%)	18 (20%)
Not at all sure	6 (13%)	5 (6%)
		
**Body mass index (BMI)**		
Underweight (Less than 18.5 kg/m^2^)	2 (4%)	0 (0%)
Normal (18.5–24.9 kg/m^2^)	20 (44%)	27 (31%)
Overweight (25.0–29.9 kg/m^2^)	15 (33%)	41 (48%)
Obese (30.0 kg/m^2 ^or higher)	8 (18%)	18 (21%)
		
*Interest in losing weight*		
Very or somewhat interested	33 (72%)	65 (73%)
A little bit or not at all interested	13 (28%)	24 (27%)

a) Percentage of available cases. Percentage values might not total 100 due to rounding.

b) p = 0.01; null hypothesis of no difference in characteristic by length of membership.

In contrast, relatively few short-term (24%) or long-term (19%) members reported consuming five or more servings of fruits and vegetables per day. The majority of short-term and long-term members were very or somewhat interested in changing their dietary behaviors (72% and 64%, respectively), were in the preparation stage of change (59% and 66%, respectively), and had a high level of self-efficacy to change their dietary habits (64% and 74%, respectively). Multivitamin use and length of membership were positively associated (p = 0.01). A majority of both short-term and long-term members were overweight or obese (51% and 69%, respectively) and were either very or somewhat interested in losing weight (72% and 73%, respectively).

We examined bivariate associations among the health behavior variables. Only one significant association was observed; among short-term members, individuals who were overweight or obese were more likely to report obtaining at least 150 minutes per week of vigorous or moderate physical activity (p < 0.05; data not shown). We also examined bivariate associations between the health behavior variables and possible sociodemographic and psychosocial correlates. As shown in Table 3, individuals who were the least interested in changing dietary habits consumed the most servings of fruits and vegetables per day (p = 0.03). Respondents with the highest BMI had the highest self-efficacy to change physical activity (p = 0.01) and the highest interest in changing dietary habits (p = 0.01). Reported YMCA usage was not significantly related to physical activity or BMI (data not shown).

**Table 3:** Bivariate associations between sociodemographic and psychosocial correlates and health behavior variables (n = 135)

	*Mean minutes/week of moderate or vigorous physical activity*	*Mean servings/day of fruits and vegetables*	*Mean body mass index (kg/m*^2^)
*Education*			
Some high school	691.3	2.4	27.0
Completed high school	2035.8	3.9	25.7
Vocational/trade school	2590.0	2.9	26.5
Some college	1293.3	2.6	28.0
Associate degree	1246.0	3.4	25.9
Bachelor degree	1072.4	3.0	27.3
Post graduate degree	1135.7	3.7	25.7
			
*Race/ethnicity*			
Black	1006.9	2.7	28.9
Hispanic	2228.3	2.6	25.8
White	1246.1	3.4	26.5
Other	1276.2	3.3	25.6
			
*Income*			
Under $20,000	1066.5	3.5	26.0
$20,000–$39,999	968.3	3.1	26.0
$40,000–$59,999	2017.3	3.2	25.3
$60,000–$79,999	1412.6	3.2	27.6
$80,000 or above	1231.3	3.3	28.2
			
*Interest in changing PA*			
Very interested	1370.8	3.0	27.1
Somewhat interested	1800.5	3.3	26.8
A little bit interested	1035.7	3.9	24.2
Not at all interested	843.0	3.7	25.7
			
*Self-efficacy to change PA*			
Sure	1353.2	3.3	26.9^a^
Maybe	1224.2	3.2	25.6
Not at all sure	1722.0	1.9	23.9
			
*Interest in changing dietary habits*			
Very interested	1434.6	2.9^b^	28.6^a^
Somewhat interested	1429.8	3.2	25.9
A little bit interested	1076.3	3.7	25.0
Not at all interested	1218.3	3.7	25.1

a) p = 0.01

b) p = 0.03

In multivariate regression models, between one and three sociodemographic or psychosocial variables entered the models as significant correlates of the health behavior outcomes. As shown in Table 4, being female was associated with having a lower BMI (p = 0.003). Females (p = 0.008), older individuals (p = 0.0004), and those with higher education (p = 0.02) reported obtaining less moderate or vigorous physical activity per week. Finally, those who consumed more servings of fruits and vegetables per day were less interested in changing their dietary behaviors (p = 0.03).

**Table 4:** Multivariate associations between sociodemographic and psychosocial correlates and health behavior variables among a sample of YMCA members (n = 135)

	Parameter estimate	p-value
*Outcome = Body mass index (kg/m*^2^)		
Intercept	27.9	
Female gender	-2.4	0.003
		
*Outcome = Physical activity (minutes/week)*		
Intercept	4143.6	
Female gender	-692.2	0.008
Age	-30.5	0.0004
Education	-165.8	0.02
		
*Outcome = Fruit and vegetable consumption (servings/day)*		
Intercept	3.8	
Interest in changing dietary behaviors	-0.3	0.03

## Discussion

The purpose of this study was to characterize health behaviors and related characteristics among YMCA members as part of examining the need for an intervention focused on weight control and associated behaviors and to provide data to help inform the development of an intervention tailored to this community-focused wellness organization. We surveyed randomly selected members to assess self-reported physical activity, dietary behaviors, and rates of overweight and obesity.

The majority of respondents reported meeting current physical activity recommendations (i.e., 150 minutes per week or more of moderate or vigorous physical activity). The high percentage of respondents meeting this physical activity recommendation is in notable contrast to national studies conducted in the general population, which have shown lower adherence rates [14-16]. This result might be expected given that the respondents were members of a fitness facility at the time of completing the survey. However, there may also have been issues with the self reports of physical activity; we did not have observational or objective data with which to corroborate the self-reported physical activity data. One possibility is that individuals were reporting time spent at the facility, rather than time spent in moderate or vigorous physical activity. The lack of an observed relationship between self-reported physical activity and YMCA usage during the previous three months also suggests that a better measure of time actually spent in moderate or vigorous physical activity at the facility is needed.

Despite the physical activity level reported by respondents, more than half were overweight or obese. The 19% rate of obesity observed among respondents is slightly lower, although consistent with, the 20–24% rate from recent statewide data in Massachusetts [17]. The relatively high prevalence of obesity observed, even among long-term members, suggests that dietary change and higher levels of physical activity might be needed for significant weight loss and control in this population. Few members reported consuming five or more servings of fruits and vegetables per day, which further indicates the need for dietary change interventions. We did not observe the hypothesized relationships between physical activity, dietary behaviors, and weight status and length of membership. These data suggest that membership in a community wellness and fitness facility might not be sufficient to help members control their weight and meet dietary recommendations, and that targeted weight control interventions may be needed. However, as mentioned above, more careful measurement of physical activity within and outside of the fitness facility is necessary to understand the observed results more completely, as is a more detailed and complete assessment of energy intake. In addition, recent data from the trial of an eight-week culturally targeted nutrition and physical activity intervention conducted in a black-owned commercial gym with healthy, obese African-American women indicated that not even free gym memberships and successful nutrition programs were enough for longer-term fitness enhancement [18,19]. These data indicated that multi-level interventions targeting physical or social/environmental contributors might be necessary for improving weight control over the long term [18].

In addition to indicating fairly high rates of overweight and obesity in this population, the survey findings suggested that respondents would be receptive to and ready for a weight control intervention. Survey respondents were generally quite interested in changing their physical activity and dietary behaviors and in losing weight. Most respondents were in the preparation stage of change with respect to physical activity and dietary behaviors and had high levels of self-efficacy to change these behaviors, suggesting that they were motivated and felt confident in their ability to change multiple risk behaviors.

The results of the survey also suggested that targeting short-term or newer members of community fitness and wellness organizations such as YMCAs might be an effective channel for reaching underserved populations. Short-term members had lower levels of education and annual household income than did longer-term members, and were also significantly less likely to self identify as white. The potential of community-based organizations such as YMCAs for effectively reaching these underserved audiences with weight control interventions should be explored with additional research.

Interestingly, although these data did indicate some significant associations between sociodemographic characteristics and health behaviors, the directions of the associations were not always consistent with what has been observed in previous research. For example, we did not observe significant associations of education, income, gender or age with fruit and vegetable consumption, as has been found in previous studies [6,20-24]. Nor did we observe associations between BMI and race/ethnicity, whereas previous studies have indicated that race/ethnicity is an important correlate of overweight and obesity in the general population [14-16]. Further study of these relationships in a larger population of members of community-focused wellness organizations is warranted. Community-based fitness facilities might be particularly successful in encouraging physical activity among individuals of color by providing safe and adequate facilities, for example, an important area for future research.

The limitations of this study are important to consider in interpreting our results. There might be important differences between respondents and non-respondents, as indicated by the less than optimal response rate, which could affect perceived need for and receptivity to a weight control intervention in this population. Less physically active individuals might have refused to participate, for example, as might those who were less motivated or who had less self efficacy to change behaviors. Physical activity, dietary behaviors, and BMI were based on self-report, although we used previously validated measures where possible. We measured physical activity with the short form of the IPAQ, which was created for national and regional surveillance systems and might not be appropriate for a small sample. In addition, social desirability bias might affect reporting of physical activity or weight, because respondents were members of a fitness facility. The motivation to change measure did not exclude individuals already meeting recommendations from the precontemplation group, as it assessed motivation for further behavior change. We did not collect data on previous fitness facility memberships or participation, so it is possible, for example, that short-term members were previously members of different health/fitness facilities or regularly exercised prior to joining this YMCA facility. The survey design was cross-sectional, so the data do not indicate whether respondents were able to maintain recommended levels of health behaviors (e.g., physical activity) consistently over time. We surveyed members of only one YMCA facility, so the results might not be generalizable to other community fitness settings. We did not assess more structural-level correlates, such as location of home or work and whether participants received free/reduced price memberships, meaning that we could not examine possible associations between these variables and individual-level correlates.

## Conclusion

This study examined health behaviors and related characteristics among both short-term and long-term members of an urban community YMCA facility. The data indicated a need for an intervention focused on weight control and related health behaviors. Although 93% of the survey respondents reported meeting current physical activity recommendations, few (21%) met fruit and vegetable consumption recommendations and more than half were overweight or obese. The findings indicated that membership in a community wellness and fitness facility might not be sufficient to help members maintain a healthy weight, and that targeted weight control and physical activity interventions are needed. The results also indicated that the YMCA members were interested in making health behavior changes and were primed to do so. The survey findings suggested that targeting newer members of community wellness and fitness organizations such as YMCAs might be an effective way of reaching underserved populations with a weight control intervention; this is an important area for research. These survey data will help inform the development of a physical activity and weight control intervention for the YMCA setting. Given the ubiquity of this organization, dissemination of future evidence-based weight control interventions through YMCAs might be an effective strategy to reach broad segments of the population and begin to reduce health disparities in chronic disease.

## Competing interests

The author(s) declare that they have no competing interests.

## Authors' contributions

KAK was responsible for designing the study, data collection, interpreting the findings and writing the paper. GGB was responsible for designing the study, interpreting the findings and writing the paper. GS was responsible for designing the study, and contributed to interpretation of the findings and critical review of the paper. KMK was responsible for data collection, and contributed to interpretation of the findings and critical review of the paper. AO was responsible for data collection and commented on drafts of this paper. KM was responsible for designing the study, data collection, and contributed to interpretation of the findings and critical review of the paper. All authors read and approved the manuscript.

## Pre-publication history

The pre-publication history for this paper can be accessed here:


